# Botulinum toxin in the management of acquired motor fusion deficiency

**DOI:** 10.4103/0301-4738.57162

**Published:** 2009

**Authors:** Ramesh Murthy, Siddharth Kesarwani

**Affiliations:** 1 Department of Pediatric Ophthalmology and Strabismus, Orbital diseases and Ocular Oncology, LV Prasad Eye Institute, Hyderabad, India.; 2 Department of Oculoplasty, Orbital diseases and Ocular Oncology, LV Prasad Eye Institute, Hyderabad, India.

**Keywords:** Acquired motor fusion deficiency, botulinum toxin

## Abstract

Acquired disruption of motor fusion is a rare condition characterized by intractable diplopia. Management of these patients is extremely difficult. Prisms in any combination or even surgery may not help relieve their symptoms. We describe a longstanding case of acquired motor fusion disruption which was managed successfully with botulinum toxin injection.

Acquired disruption of motor fusion is characterized by diplopia in all positions of gaze and is associated with strabismus.[1] It can occur following prolonged sensory deprivation in one eye as can occur when a previously normal eye has an untreated monocular cataract for a prolonged period or sometimes even after cataract extraction.[1,2] It can occur after severe head injury, postviral syndromes or with other central nervous system disorders.[1] Patients with this disorder experience constant diplopia that cannot be eliminated with prisms. As they have lost central fusional abilities they can neither fuse nor suppress the images. In this report we describe a patient who developed acquired motor fusion deficiency which was managed by botulinum toxin injection with a successful outcome.

## Case Report

**Figure 1:**
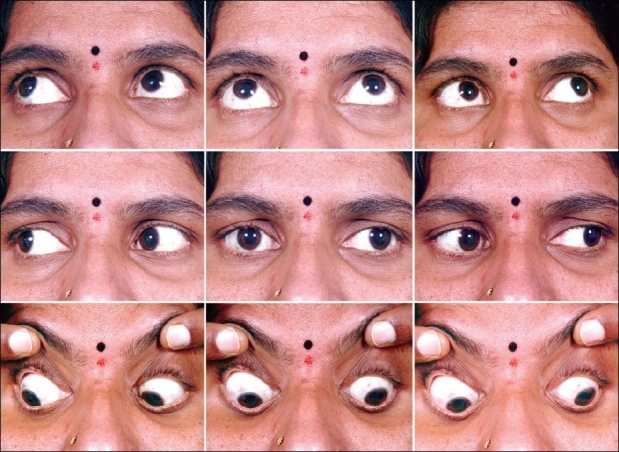
Clinical photograph pre injection showing the presence of comitant left exotropia in all the nine cardinal positions of gaze

**Figure 2:**
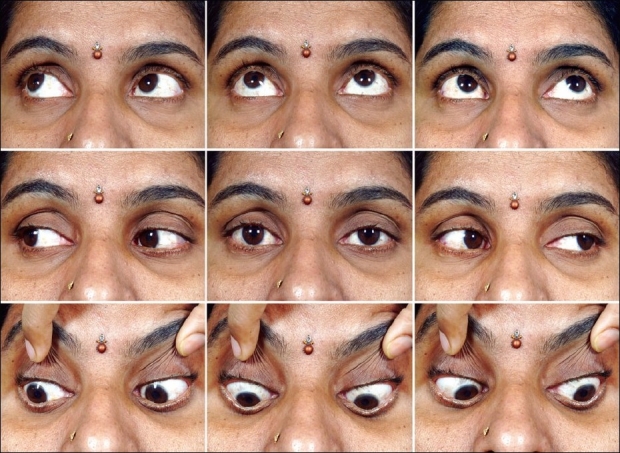
Clinical photograph six months after the final injection of botulinum toxin demonstrating well-aligned eyes in all the nine cardinal positions of gaze

A 30-year-old lady presented to the squint clinic with complaints of constant diplopia and outward deviation of the right eye following head trauma three years back in a road accident. Prior to the accident, she had never had diplopia or squinting of the eyes. She had developed bilateral sensorineural hearing loss following the accident. Computed tomography (CT) scan had revealed a subdural hematoma in the right frontal region which had been managed conservatively and had resolved. On examination her visual acuity was 20/20 for distance and N6 for near in each eye. Anterior segment and fundus examination was within normal limits. There was no sign of any macular disease on fundus examination. Cover testing revealed a freely alternating exodeviation of 40 prism dioptres (PD) for near and distance [Fig. 1]. Convergence could not be demonstrated. Ductions were full in all gazes. Diplopia charting revealed binocular crossed diplopia with constant separation of images in all gazes. There was no indication of torsional abnormality on diplopia testing. Humphrey visual field testing did not reveal any field loss. Fusion was not possible in free space with any combination of prisms even after prolonged prism adaptation in the clinic on two consequent visits. Based on this a diagnosis of acquired motor fusion deficiency was made. She was administered botulinum toxin injection (Botox, Allergan Inc) 2.5 units into each lateral rectus muscle under electromyographic guidance. Review at the end of one month revealed a reduction in the exotropia to 10 PD and subjective appreciation of closeness of the two images. She was subsequently administered a repeat injection of 2.5 units of botulinum toxin into the left lateral rectus muscle. Follow-up after one month revealed complete resolution of the diplopia. There was no abnormal head posture. Objective examination revealed she was orthophoric for near and exophoric for distance and extraocular movements were full. Follow-up six months later revealed she was completely symptom-free. There was no exotropia on cover test [Fig. 2]. The horizontal fusion ranges were 30 PD base out and 16 PD base in for near and 16 PD base out and 8 PD base in for distance. The vertical fusion range was 3 PD base up and 3 PD base down. She demonstrated gross stereopsis on the Wirt fly test.

## Discussion

Acquired strabismus occurring after visual maturation results in diplopia. In most cases the ability to fuse allows the patients to regain binocular single vision when the misalignment is eliminated.[3,4] However, in patients with central disruption of fusional amplitude, the ability to superimpose the images may be completely lost. This results in constant diplopia which can be eliminated only by occluding one eye.[5] A diagnosis of central disruption of fusion should not be made unless all other causes for intractable diplopia have been excluded, including torsion and aniseikonia.[1,6] Prolonged prism adaptation or testing on a synoptophore is essential before making this diagnosis.

Strabismus is common after head trauma, even if the brainstem is not obviously affected. The strabismus may be due to paresis of one or more cranial nerves, diffuse brain damage or due to central disruption of fusional amplitudes because of damage to the vergence centers of the midbrain.[2] Sometimes the brain may simply lose its ability to fuse because of prolonged deprivation of fusable images.[2] Pratt-Johnson hypothesized that midbrain damage may be a cause for the disruption of convergence and or divergence, leading to inability to fuse.[5] Orthoptic treatment has been reported to be effective in patients where there is a diminished fusional range without a complete loss of fusion.[7] However, this may not happen in cases where there is no demonstrable fusion.

Consequently, if the lack of fusable images can lead to disruption of central fusion as following prolonged occlusion due to cataract, presence of fusable images may aid in recovery from central fusion disruption. In our patient botulinum toxin injections helped to align the eyes for prolonged periods thereby presenting continuous fusable images to the brain. This may have facilitated recovery from the diplopia which may or may not have resolved otherwise. Botulinum toxin is a minimally invasive, quick outpatient procedure and can be repeated. Botulinum toxin may be worth attempting in patients with intractable diplopia following central fusion disruption.
